# The effect of lipoprotein-associated phospholipase A_2_ deficiency on pulmonary allergic responses in *aspergillus fumigatus* sensitized mice

**DOI:** 10.1186/1465-9921-13-100

**Published:** 2012-11-12

**Authors:** Zhilong Jiang, Melane L Fehrenbach, Giulia Ravaioli, Blerina Kokalari, Imre G Redai, Steven A Sheardown, Stephen Wilson, Colin Macphee, Angela Haczku

**Affiliations:** 1 Pulmonary, Allergy and Critical Care Division, University of Pennsylvania, Philadelphia, PA, USA; 2 Takeda Cambridge Limited, 418 Cambridge Science Park, Cambridge, UK; 3 GSK Laboratory Animal Sciences, GlaxoSmithKline, Stevenage, UK; 4 Department of Vascular Biology and Thrombosis, GlaxoSmithKline, King of Prussia, PA, USA; 5 Pulmonary, Allergy and Critical Care Division, Translational Research Laboratories, 125 South 31st Street, Philadelphia, PA, 19104-3403, USA

**Keywords:** Lp-PLA_2_, PAF-AH, Knock-out mice, Airway inflammation, IgE, Mast cells, Degranulation

## Abstract

**Background:**

Lipoprotein-associated phospholipase A_2_ (Lp-PLA_2_)/platelet-activating factor acetylhydrolase (PAF-AH) has been implicated in the pathogenesis of cardiovascular disease. A therapeutic targeting of this enzyme was challenged by the concern that increased circulating platelet activating factor (PAF) may predispose to or increase the severity of the allergic airway response. The aim of this study was to investigate whether Lp-PLA_2_ gene deficiency increases the risk of PAF and IgE-mediated inflammatory responses *in vitro* and *in vivo* using mouse models.

**Methods:**

Lp-PLA_2_-/- mice were generated and back crossed to the C57BL/6 background. PAF-AH activity was measured using a hydrolysis assay in serum and bronchoalveolar lavage (BAL) samples obtained from mice. *Aspergillus fumigatus* (*Af*)-specific serum was prepared for passive allergic sensitization of mice *in vivo* and mast cells *in vitro*. β- hexosaminidase release was studied in bone marrow derived mast cells sensitized with *Af*-specific serum or DNP-IgE and challenged with *Af* or DNP, respectively. Mice were treated with lipopolysaccharide (LPS) and PAF intratracheally and studied 24 hours later. Mice were sensitized either passively or actively against *Af* and were studied 48 hours after a single intranasal *Af* challenge. Airway responsiveness to methacholine, inflammatory cell influx in the lung tissue and BAL, immunoglobulin (ELISA) and cytokine (Luminex) profiles were compared between the wild type (WT) and Lp-PLA_2_-/- mice.

**Results:**

PAF-AH activity was reduced but not completely abolished in Lp-PLA_2_-/- serum or by *in vitro* treatment of serum samples with a high saturating concentration of the selective Lp-PLA_2_ inhibitor, SB-435495. PAF inhalation significantly enhanced airway inflammation of LPS treated WT and Lp-PLA_2_-/- mice to a similar extent. Sensitized WT and Lp-PLA_2_-/- bone-marrow derived mast cells released β-hexosaminidase following stimulation by allergen or IgE crosslinking to equivalent levels. Wild type and Lp-PLA_2_-/- mice responded to passive or active allergic sensitization by significant IgE production, airway inflammation and hyperresponsiveness after *Af* challenge. BAL cell influx was not different between these strains while IL-4, IL-5, IL-6 and eotaxin release was attenuated in Lp-PLA_2_-/- mice. There were no differences in the amount of total IgE levels in the *Af* sensitized WT and Lp-PLA_2_-/- mice.

**Conclusions:**

We conclude that Lp-PLA_2_ deficiency in C57BL/6 mice did not result in a heightened airway inflammation or hyperresponsiveness after PAF/LPS treatment or passive or active allergic sensitization and challenge.

## Introduction

Lipoprotein-associated phospholipase A_2_ (Lp-PLA_2_) is a 45-kDa protein of 441 amino acids encoded by the *pla2g7* gene in humans. In the blood it travels mainly with low density lipoprotein (LDL) and less than 20% is associated with high density lipoprotein (HDL). This enzyme is produced by myeloid derived cells and it functions to hydrolyze oxidized/polar phospholipids. Whether Lp-PLA_2_ is a pro- or anti-inflammatory mediator is the subject of intense debate and numerous studies involving clinical trials and animal models [1].

Lp-PLA_2_ is implicated in the development of atherosclerosis [2]. A meta-analysis on a total of 79,036 participants in 32 prospective studies found that serum Lp-PLA_2_ positively correlated with an increased risk of coronary heart disease and stroke [3]. In atherosclerotic lesions the main sources of Lp-PLA_2_ include LDL from the circulation, and *de novo* synthesis by the inflammatory cells found in the plaque (macrophages, platelets, mast cells) [4]. Products of Lp-PLA_2_ can upregulate expression of adhesion molecules, activate leucocytes and recruit macrophages and monocytes into inflammatory areas [5-7]. Inhibition of Lp-PLA_2_ by the highly potent and selective inhibitor darapladib effectively ameliorated the clinical severity of atherosclerosis and deceased inflammation in the plaque area in a swine model [8]. Therefore, targeting of Lp-PLA_2_ has become an attractive strategy for the treatment of atherosclerosis.

Lp-PLA_2_ is also called platelet-activating factor acetylhydrolase (PAF-AH), as it can cleave platelet-activating factor (PAF) *in vitro* by hydrolysis of the acetyl group at the sn-2 position, producing lyso-PAF and acetate [9,10]. PAF plays a prominent role in the pathogenesis of IgE mediated allergic inflammation and anaphylaxis (reviewed in [11-15]). Therapeutic targeting of PAF however did not affect asthma symptoms [16]. Nonetheless because of its PAF catalyzing activity [17-22], inhibition of PAF-AH/Lp-PLA_2_ raised the concern of an increased predisposition to allergic inflammation or anaphylaxis. Although the published direct evidence to support this concern is limited, there were clinical associations reported between low PAF-AH/Lp-PLA_2_, high plasma PAF and increased incidents and severity of asthma [23-26] and anaphylaxis [19]. A single nucleotide polymorphism of Val-279-Phe in the PAF-AH/Lp-PLA_2_ gene with functional deficiency was shown to be highly prevalent in Japan (about 4% of the general Japanese population) [27]. According to a 1999 study by Stafforini et al. PAF-AH/Lp-PLA_2_ deficiency was increased in asthmatics in comparison with healthy subjects in Japan with the greatest asthma severity found in homozygous PAF-AH/Lp-PLA_2_ deficient subjects [25]. In animal models of lung injury and sepsis elevated PAF-AH/Lp-PLA_2_ levels were reported to be associated with inhibitory effects during the acute inflammatory process [28,29]. Exogenous administration of PAF-AH/Lp-PLA_2_ reduced mortality [18] and over-expression of PAF-AH/Lp-PLA_2_ attenuated inflammation in mouse models of sepsis [17,18,30] suggesting that this enzyme may have protective effects against inflammatory mechanisms involving PAF.

This suggestion was contested in a clinical study conducted very similarly to that of Stafforini’s. In this work Satoh and colleagues found no difference in the allele frequency between asthmatic patients and healthy controls and the V279F mutant allele prevalence was consistent regardless of asthma type or severity of disease [31]. In a more recent study Japanese patients underwent a bronchoprovocation test with PAF showed no difference in airway responsiveness whether they had the V279F mutant allele or not [32]. Further, in human studies treatment with a recombinant PAF-AH/Lp-PLA_2_ preparation showed no sufficient efficacy in asthma or in sepsis ([33] reviewed in [1]).

Based on these controversial data, the question whether low circulating PAF-AH/Lp-PLA_2_ may predispose to heightened inflammation and IgE-mediated allergic immune responses remains to be clarified. The aim of our study was to investigate whether genetic targeting of PAF-AH/Lp-PLA_2_ would result in pro- or anti-inflammatory changes following allergen exposure in a murine model of allergic sensitization.

## Methods

### Mice

Wild type C57BL/6 mice (6 to 8 weeks old) were purchased from the Jackson Laboratories (Bar Harbor, ME). Wild-type mice and homozygous Lp-PLA_2_-/- mice have been maintained in specific pathogen-free facilities at the University of Pennsylvania. Mice were fed chow and water ad libitum and were under a 12 hour daylight/dark cycle. The University of Pennsylvania Institutional Animal Care and Use Committee (IACUC) approved all protocols, and all experiments were performed in accordance with the guidelines of the University of Pennsylvania IACUC.

### The Lp-PLA_2_ targeting strategy

**Figure 1:**
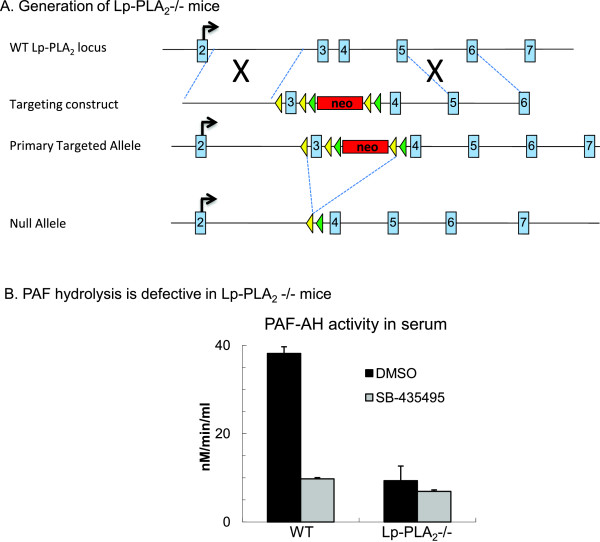
**Lp-PLA**_**2**_**targeting strategy and measurement of PAF-AH activity in mice.** (**A**): Generation of Lp-PLA_2_-/- mice: Homologous recombination in ES cells was used to generate ES cells heterozygous for the Lp-PLA_2_ primary targeted allele. Breeding of mice heterozygous for the Lppla2 primary targeted allele with a germline Cre-deleter strain resulted in mice heterozygous for the Lp-PLA_2_ null allele. Lp-PLA_2_ exons are indicated as blue boxes; the neo^**r**^ selection cassette as a red box; loxP and FRT sites as yellow and green arrowheads respectively. The positions of the PCR-derived homology arms in relation to the genomic locus structure are indicated by dotted lines. (**B**): PAF hydrolysis is defective in Lp-PLA2-/- mice. PAF-AH activity in the serum of C57BL/6 wild type (WT) and Lp-PLA2-/- mice was measured as described. Data are expressed as Mean±SEM; n=6-8.

Standard gene targeting approaches were used to generate 129-C57BL/6J hybrid ES cells heterozygous for the Lp-PLA_2_ primary targeted allele, which contains two loxP sites flanking a neo cassette within intron 3 and an additional loxP site within intron 2. 5′ and 3′ homology arms (3240bp and 4496bp respectively) and the ‘floxed’ exon 3 region (872bp) were isolated by PCR from C57BL/6J genomic DNA (Figure 1A). Homologous recombination in neomycin resistant ES cells was confirmed at the 5′end by Southern blot analysis using probes external to the homology arm (data not shown), and at the 3′ end by long range PCR using primers internal and external to the targeting vector. Two correctly targeted ES cell clones were injected into Balb/c-derived blastocysts, and resultant male chimaeras were crossed with C57BL/6J females to produce mice heterozygous for the Lp-PLA_2_ primary targeted allele. These were subsequently bred to a germline Cre- deleter strain resulting in mice heterozygous for the Lp-PLA_2_ null allele (Lp-PLA_2_n/+). Marker-assisted backcrossing was then employed to achieve a genetic background >98% C57BL/6J. Study populations were produced by breeding the homozygous strains.

### PAF acetyl hydrolase activity

This was performed as previously described [34]. Briefly, Lp-PLA_2_ activity was measured using either 1-decanoyl-2-(4-nitrophenylglutaryl) phosphate (DNGP) or ^3^H]PAF (Cascade Biochemicals) as a substrate. All assays were performed at 37°C in 50 mmol/L HEPES and 150 mmol/L NaCl, pH 7.4. Serum samples (at different dilutions) were added to 50 μmol/L DNGP in buffer at 37°C. The absorbance increase was followed at 400 nm, using either a diode array spectrophotometer (Hewlett-Packard) or a 96-well plate reader (Molecular Devices, Tmax) running in kinetic mode. Product was quantified using the published extinction coefficient, ε_400_=15 000·L·mol^-1^·cm^-1^[34]. For PAF-AH activity, ^3^H]PAF and sample were incubated in a final volume of 200 μL for 10 minutes at 37°C. The reaction was stopped by vortexing with 600 μL of CHCl_3_/MeOH (2:1), and the CHCl_3_ and aqueous layers were separated by centrifugation. The aqueous layer was removed (250 μL) and vortexed with 250 μL of CHCl_3_. The aqueous layer was again removed and the ^3^H]acetate determined by scintillation counting. Protein was determined using the Pierce bicinchonic acid assay kit, according to the manufacturer's instructions. To determine the inhibitory effect of SB-435495, the serum samples and the compound were preincubated at 37°C for 10 minutes before running the enzyme assay as described above (Figure 1B).

### LPS/PAF treatment

Following experiments to determine the optimal dose and time course, C57BL/6 wild type (WT) and Lp-PLA_2_-/- mice (n=6-7 in each group) were administered 1 μg LPS and 1 μg of a 1:1 mixture of PAF (C16) and PAF (C18) *via* intra-tracheal instillation under light ketamine/xylazine anesthesia. 24 hours later lung function measurements were performed and bronchoalveolar lavage (BAL) was obtained from each mice as described below.

### Preparation of *Aspergillus fumigatus* (*Af*)-specific serum for passive sensitization

Wild type male C57BL/6 mice (n=10) at 12-15 weeks of age, were injected intraperitoneally (i.p.) with 20 μg *Af* adsorbed on alum, on day 1 and 7 and then received intranasal (i.n.) treatment with 30 μg *Af* on day 13 and subsequently every other days to total 6 doses. Mice were sacrificed 2 days after the last allergen treatment and blood was obtained in vacutainers. Blood from naïve mice (n=10) was obtained for naive serum. Serum samples were pooled, aliquoted and stored at -20ºC.

Total IgE was measured by a mouse IgE ELISA kit (GenWay, San Diego CA) according to manufacturer’s instructions. *Af*-specific immunoglobulin levels were determined as previously described [35]. Serum (dilution 1:100) or undiluted BAL supernatant samples were loaded from individual mice.

### Mast cell culture and function assay

**Figure 2:**
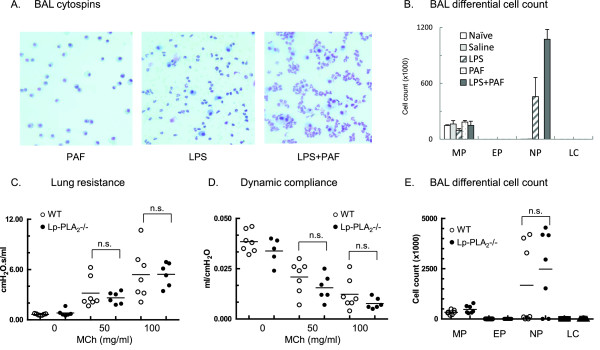
**Lp-PLA2 deficiency did not enhance the function of bone marrow-derived mast cells.** (**A**): Bone marrow cells harvested from C57BL/6 wild type (WT, white bars) and Lp-PLA2-/- (black bars) mice were cultured for 3-4 weeks in the presence of 20 ng/ml murine IL-3. Mast cells were identified by phase contrast microscope (top panels) and FACS analysis using antibodies against FcεRI and CD117 (bottom panels). (**B**): Cells were sensitized with pooled WT anti-Af serum (1:100 dilution) and challenged with 3 μg/ml Af in vitro (n=4, left panel). Hexosaminidase release was measured in non-treated cells, cells treated with naïve serum alone or anti-Af serum alone (not shown); naïve serum+Af and anti-Af serum+Af. Anti-DNP IgE (2 μg/ml) and DNP (25 μg/ml) treated cells were used as positive controls (n=4). Data presented as % of the total β-hexosaminidase present in 0.1% Triton X-100 lysed cells. (**C**): Cells derived from wild-type or LP-PLA2-/- mice were sensitized with individual anti-Af serum samples, derived from WT mice that received active sensitization (n=9-10). Cells then were challenged with 3 μg/ml Af. Median and interquartile ranges. The assay was performed in triplicates (left panel). The right panel shows a correlation between Lp-PLA2 levels (as measured by PAF-AH activity) and mast cell degranulating activity (hexosaminidase release) of 6 serum samples. Data are presented as ratio over naïve serum control. (B-C): Released hexosaminidase in supernatant was measured by reaction with 8 mM p-nitrophenyl-N-acetyl-β-d-glucopyranoside (PNP-NAG). Data calculated from the total β-hexosaminidase present in 0.1% Triton X-100 lysed cells.

Mast cell function was assessed using bone-marrow derived mast cells and hexosaminidase release in response to sensitization with specific anti-*Af* serum or anti-DNP IgE antibody (Sigma, St. Louis, MO). Bone marrow was harvested from wild-type C57BL/6 mice (The Jackson Laboratory, Bar Harbor, ME) and Lp-PLA_2_-/- mice. Cells were then purified on ficoll-paque (Amersham Biosciences, Sweden) and maintained for 3-4 weeks in RPMI1640 medium supplied with 20 ng/ml murine IL-3. Mast cells were characterized by morphology under phase contrast microscope (Figure 2A upper panels) and FACS analysis using antibodies against FcεRI and CD117 (BD Pharmingen, Figure 2A lower panels). The cells were sensitized with mouse serum containing IgE against *Af* (1:100 dilution) for 12 hr and challenged with 3 μg/ml *Af* for 2 hr. Anti-DNP IgE (2 μg/ml) and DNP (10 μg/ml) were used as positive control. Cells were either sensitized alone or challenged alone as negative controls. Hexosaminidase released from activated cells was measured by reaction with 8 mM *p*-nitrophenyl-*N-*acetyl-β-d-glucopyranoside (PNP-NAG, Sigma) for 12 hours, optical density (OD) was measured on 405 nm spectermeter. Data was presented as fold increase over naïve cell culture medium.

### Passive allergic airway sensitization using *Aspergillus fumigatus* (*Af*)-specific serum

This sensitization model is based on the effects of allergen specific immunoglobulins and other mediators in pooled serum of actively sensitized mice. Inflammation is thought to be mediated by interactions between immunoglobulin IgE and the mast cell high affinity IgE receptor FcεR-I, leading to mast cell degranulation and release of a variety of inflammatory mediators. To elicit the IgE-mediated changes, 9-12 week-old male WT and Lp-PLA2-/- mice were sensitized i.v. by anti-*Af* specific serum on day 1, day 2 and day 3. Dose-response studies determined that the optimal serum dilution to elicit the expected inflammatory changes fell between 1:100 and 1:1000 (not shown). We used a dilution of 1:200 throughout the study. 30 μg *Af* was applied intranasally to mice on day 5. Mice were studied 72 hours after *Af* challenge. Negative control mice received naive serum and/or were challenged with PBS.

### Active allergic airway sensitization with *Af*

For active allergic sensitization, 9-12 week-old male C57BL/6 wild-type and Lp-PLA2-/- mice were injected intraperitoneally (i.p.) with 20 μg of *Af* pre-formulated with alum (volume 1:1) on day 1 and 7 and then challenged intranasally (i.n.) with 30 μg *Af* on day 13. Mice were studied on day 15.

### Lung function measurements

Lung function was measured as described previously [36-40]. Airway hyperresponsiveness to methacholine (MCh) inhalation was assessed using the FlexiVent system (Sireq, Montreal, Canada). This method is suitable to measure lung resistance and dynamic compliance, the most important physiological characteristics of the inflammatory airway response. Briefly, lung mechanics were studied in tracheostomized mice under anesthesia by intra-peritoneal injection of ketamine and xylazine. Mice were ventilated with a tidal volume of 8ml/kg at a rate of 150 breaths/min and a positive end-expiratory pressure of 2cm H_2_O by a computerized FlexiVent System. After mechanical ventilation for 2 min, a sinusoidal 1-Hz oscillation was applied to the tracheal tube. The single-compartment model was fitted to these data by multiple linear regression to calculate dynamic resistance and compliance of the airway. At the end of lung function measurement, BAL cells were collected for cytospin preparation and differential cell counting. The BAL supernatant was aliquoted and stored at -80°C.

### Inflammatory profile

Lung inflammatory profile was assessed as described previously in our laboratory [35,36,41-44]. To assess activation of inflammatory cells, serum and BAL were obtained at the end of experiments. Measurements of cytokines/chemokines were performed using Searchlight technology (Aushon Biosystems Inc, Billerica MA).

### Statistical analysis

The number of mice used in the study was determined by standard power calculations using preliminary data and our previous experience in these models. The number of mice used in individual groups for statistical analysis is stated in the figure legends. Results are presented as the mean ± standard error (SE) of each group. Unless otherwise stated, data were analyzed by two-way ANOVA followed by a Bonferroni post-hoc test. A value of *p* < 0.05 was considered as statistically significant. All statistical analyses were performed using Graphpad Prism software (GraphPad Software Inc, La Jolla, CA).

## Results and discussion

### PAF-AH activity is reduced but not abolished by *in vitro* treatment with high concentration of SB-435495 and in Lp-PLA2-/- mice

To determine whether PAF-AH/Lp-PLA_2_ activity is altered by the enzyme inhibitor SB-435495 in the mouse serum, samples from wild type C57BL/6 and Lp-PLA_2_-/- mice were investigated using a previously established technique based on a modified hydrolysis assay [45]. This assay measures the enzyme product lyso-PAF.

The average serum PAF-AH activity was 38.1 and 9.3 nM/min/ml in naive WT and Lp-PLA_2_-/- mice, respectively. Treatment of WT serum with 10μM of SB-435495 (a saturating concentration used to completely abolish Lp-PLA_2_), reduced enzyme activity to levels observed in serum from Lp-PLA_2_-/- mice lacking a functional Lp-PLA_2_ gene (Figure 1A-B). In both WT and Lp-PLA_2_-/- mice there was a significant residual PAF-AH activity in the serum which was obviously not attributable to Lp-PLA_2_. These findings suggest that Lp-PLA_2_ may not be the exclusive PAF-cleaving enzyme. Further, it is likely that the main mechanism of action of this enzyme is not the cleavage of PAF. Additional support for this is provided by a recent study in which treatment of mice with darapladib (an Lp-PLA2 inhibitor) inhibited serum IL-6 levels but did not alter serum PAF levels in a model of atherosclerosis [46]. Nonetheless, in the serum of Lp-PLA_2_-/- mice PAF metabolism was significantly reduced. The *in vivo* significance of the lack of Lp-PLA_2_ has not been determined before in gene deficient animals, therefore we next investigated the effects of PAF treatment in these mice.

### LPS+PAF-induced inflammatory cell influx and airway hyperresponsiveness was similar between Lp-PLA_2_-/- and WT mice

**Figure 3:**
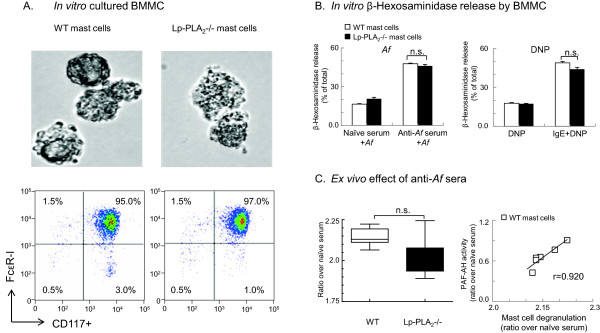
**LPS/PAF-induced inflammatory cell influx and airway hyperresponsiveness was comparable between Lp-PLA**_**2**_**-/- and WT mice.** (**A**-**B**): C57BL/6 mice were administered nothing (Naïve, white bars), saline (light grey bars), 1 μg LPS (hatched bars), 1 μg PAF (dotted bars) or 1 μg LPS+PAF (dark grey bars) via intra-tracheal instillation under light anesthesia. BAL was obtained 24 hours later. (**A**): Cytospin preparations of the cell pellet were assessed under light microscope. Giemsa stained panels are showing representative BAL samples from PAF (left panel), LPS (middle panel) and LPS+PAF (right panel) treated mice. (**B**): Differential cell count was performed using total cell counts and cytospin preparations. Cells were identified as Macrophages (MP), eosinophils (EP), neutrophils (NP) and lymphocytes (LC). Data are presented as absolute cell number of BAL (mean± SEM, n=6). (C-E): C57BL/6 wild type (WT, white circles) and LP-PLA2-/- (black circles) mice were administered 1 μg LPS+PAF via intra-tracheal instillation under light anesthesia. Lung resistance (**C**) and lung dynamic compliance (**D**) were measured by FlexiVent in response to increasing doses of MCh 24 hours after treatment. (**E**): BAL cell pellet was assessed and data are presented as absolute cell number of BAL (individual data points [n=6-7] are shown with the median indicated by a horizontal line).

PAF has been implicated in inflammatory conditions via diverse biological pathways both as an anti-inflammatory [47] and as a proinflammatory [48] mediator. Studies in mice and gerbils suggested that increased PAF levels and decreased PAF-AH activity significantly contribute to severity of inflammation in sepsis and that treatment with recombinant PAF-AH inhibit the inflammatory changes [48]. Results of human trials using recombinant PAF-AH to treat sepsis were however controversial [33]. Further, although PAF-AH/Lp-PLA_2_ can hydrolyze PAF *in vitro* (Km value for Lp-PLA_2_ for PAF is >10μM), there is little to no evidence that this enzyme is involved in the metabolism of PAF *in vivo* (where PAF is active at low nM levels). To investigate the role of PAF cleavage in airway inflammation we generated PAF-AH/Lp-PLA_2_ deficient mice on a C57BL/6 background and used a model of LPS/PAF inhalational treatment. The model was established in wild type C57BL/6 mice that were administered 1 μg LPS and a 1:1 mixture of PAF (C16) and PAF (C18) *via* intra-tracheal instillation. When instilled on its own PAF did not induce inflammatory changes however it significantly enhanced the effects of LPS on neutrophil influx (Figure 3A-B). Figure 3B shows results from experiments performed to compare the effects of PAF, LPS and the combination of the two in order to establish the optimal doses and time course of the inflammatory effect. The effects of PAF were time and dose dependent with doses 5-10 μg being lethal when combined with LPS (not shown). The greatest changes (but no lethality) were induced by the 1 μg dose 24 hours after instillation. Measurements of lung resistance, dynamic compliance and total and differential cell counts at the 24 hour time point revealed that Lp-PLA_2_ deficiency did not enhance the PAF effects on airway neutrophilia and hyperresponsiveness in this model (Figure 3C-E). Figure 3E compares WT and Lp-PLA2-/- mice treated with a combination of PAF and LPS in their BAL cell counts. A higher neutrophil count (in comparison with Figure 3B) may reflect the effects of additional lung injury due to the lung function measurements that were performed in these mice upon methacholine challenges for approximately 1 hour before sacrificed.

These results indicated that lack of PAF-AH/Lp-PLA_2_ did not increase susceptibility to the effects of exogenously administered PAF on LPS-induced airway neutrophilia and hyperresponsiveness in the gene deficient mice. In contrast to previous findings in animal models of sepsis [18,48], the data presented here suggested no protective role of PAF-AH/Lp-PLA_2_ in cellular inflammation.

Lp-PLA_2_ deficient bone marrow-derived mast cells had similar morphology, FcεR-I expression and released hexosaminidase to a similar degree to wild-type mast cells *in vitro*

In addition to sepsis, PAF is a major player in allergic inflammation. Produced by activated mast cells during the immediate allergic response, PAF is capable of amplifying mast cell activation through the PAF receptor [49]. Mast cells also produce Lp-PLA_2_ that was suggested to decrease PAF and increase lysoPAF production in an autocrine/paracrine fashion [50,51]. Lack of Lp-PLA_2_ was therefore suggested to predispose to IgE-mediated mast cell degranulation, a hallmark of severe allergic reactions such as anaphylaxis [52].

To study the effects of Lp-PLA_2_ deficiency in mast cell differentiation and degranulation we used bone marrow-derived cultures of mast cells from WT and Lp-PLA2-/- mice. At the end of the culture period mast cells from both strains of mice showed similar presence of granules in cytoplasm and had similarly high expression of FcεRI, the high affinity IgE receptor (Figure 2A). In comparison with cells treated with either the antibody or the antigen alone, mast cells after sensitization (with anti-*Af* serum or anti-DNP IgE) followed by cross-linking (with *Af* or DNP, respectively), released significant amounts of hexosaminidase, a granule product (Figure 2B). Our results demonstrate an average of 50%: a 2.5 fold increase in β-hexosaminidase release in response to *Af* when cells were sensitized with anti-*Af* serum in comparison with cells sensitized with naïve serum or, in response to DNP when cells were sensitized with anti-DNP IgE in comparison with cells treated with DNP alone. This indicates a significant degree of mast cell degranulation induced by FcεR-I crosslinking. There was a trend towards reduced degranulation by Lp-PLA_2_-/- mast cells when stimulated with anti-DNP-IgE+DNP (Figure 2B left panel). However the extent of hexosaminidase release in response to sensitization with the pooled anti-*Af* serum preparation and *Af* challenge (Figure 2B right panel) or to the treatment with individual serum samples obtained from mice in the active sensitization protocol followed by *Af* challenge (Figure 2C), was similar. Thus, cells from WT and PAF-AH/Lp-PLA_2_ deficient mice showed similar morphology, expressed FcεR-I at the same level and released hexosaminidase in response to IgE crosslinking to a similar degree. These data indicate that lack of Lp-PLA_2_ did not enhance mast cell function *in vitro*.

It is possible that gene knockout of Lp-PLA2/PAF-AH activity in mice will not generate the same types of immune/inflammatory changes as those found in humans. However, in a recent article by Dyer et al. it was shown that both human and mouse eosinophils degranulated in response to PAF or lysoPAF in a PAFR independent manner suggesting that relevant proinflammatory actions maybe mediated by both PAF and its PAF-AH induced metabolite. Thus, Lp-PLA_2_ may not necessarily play a negative feedback role during mast cell activation. Indeed, the degree of degranulation in WT mast cells positively correlated with the extent of PAF-AH/Lp-PLA_2_ expression in the serum used for sensitization of these cells (Figure 2C right panel), suggesting a possible enhancing rather than inhibitory effect on this proinflammatory mast cell function.

### Lp-PLA_2_ deficiency did not increase airway inflammation and hyperresponsiveness after allergen challenge of passively sensitized Lp-PLA2-/- mice

**Figure 4:**
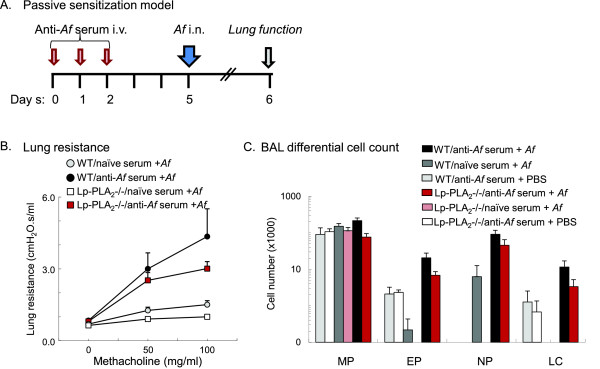
**Lp-PLA**_**2**_**deficiency did not increase airway inflammation and hyperresponsiveness after allergen challenge of passively sensitized Lp-PLA2-/- mice.** (**A**): 9-12 week-old male C57/B6 wild-type and Lp-PLA2-/- mice were sensitized i.v. by different dilutions of serum (results using 1:200 are shown) containing IgE against *Aspergillus fumigatus* (*Af*) on day 1, day 2 and day 3, then intranasally challenged with 30 μg *Af* on day 5. Lung responses were analyzed on day 6. Control mice received naive serum and/or were challenged with PBS. (**B**): Lung resistance was measured by FlexiVent in response to increasing doses of MCh. (**C**): BAL was obtained and cytospin preparations of the cell pellet were assessed under light microscope. Differential cell count was performed using total cell counts and Giemsa-stained cytospin preparations. Cells were identified as macrophages (MP), eosinophils (EP), neutrophils (NP) and lymphocytes (LC). (Mean±SEM n=5-11).

To investigate the role of Lp-PLA2 in IgE-mediated mechanisms we used a passive sensitization method previously established in our laboratory [53,54]. Using different dilutions of serum containing *Af*-specific antibodies from 1:10 to 1:100,000 we were able to elicit inflammatory changes following *Af* challenge of the mice. The optimal induction of airway hyperresponsiveness occurred at serum dilution of 1:200 (data not shown). Lung resistance and lung dynamic compliance were measured by the FlexiVent technique in response to increasing doses of inhaled methacholine. Lung resistance (RL; Figure 4B) and dynamic compliance (not shown) were not significantly different between WT and Lp-PLA_2_-/- mice albeit a trend for attenuation was observed in the latter.

Airway inflammation was further quantitated by assessing cellular composition. The total cell count in the BAL was significantly increased in sensitized and challenged mice compared to the ones that received either serum+PBS or PBS+*Af* treatment. Although a slight decrease was observed in this group, Lp-PLA_2_ deficiency did not result in significantly altered inflammatory cell influx (macrophages, eosinophils, neutrophils, lymphocytes) into the BAL fluid (Figure 4C). Although the question whether the presence and direct involvement of mast cells could be demonstrated in airway inflammation in our mouse model *in vivo* is important, this exceeded the limitations of this current study. Our results nonetheless indicate that Lp-PLA_2_ deficiency did not affect airway hyperresponsiveness and inflammatory response in the passively sensitized mice suggesting that mast cell degranulation and downstream pathologies may not be significantly affected by lack of Lp-PLA_2_. We next assessed whether Lp-PLA_2_ deficiency would impact allergic immune responses and pulmonary function in actively sensitized mice.

Lp-PLA_2_-/- mice had similar degree of airway inflammation and hyperresponsiveness, circulating and local IgE but attenuated IL-4 and IL-5 release in the BAL fluid when compared with WT mice.

**Figure 5:**
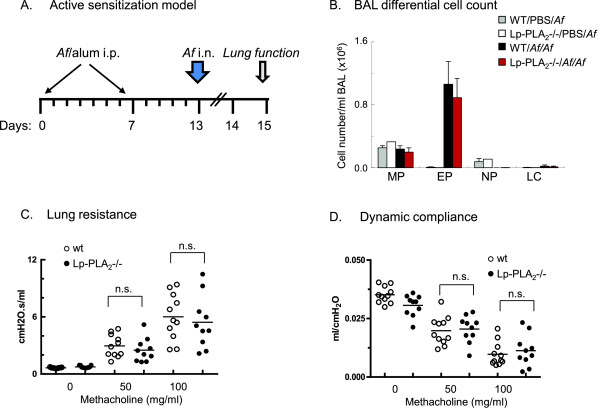
**Lp-PLA**_**2**_**deficiency did not increase inflammatory cell influx and airway hyperresponsiveness after allergen challenge of actively sensitized Lp-PLA2-/- mice.** (**A**): 9-12 week-old male C57/B6 wild-type and Lp-PLA_2_-/- mice were i.p. injected with 20 μg *Af* pre-formulated with alum (volume 1:1) on day 1 and 7, then intranasally challenged with 30 μg *Af* on day 13 and studied on day 14 or 15. Mice received *Af* challenge alone were negative controls. (**B**): Macrophages, eosinophils, neutrophils, lymphocytes in BAL were counted after cytospin preparations and Giemsa staining. Data are presented as absolute cell number of BAL (mean± sem, n=10-11). Lung resistance (**C**) and lung dynamic compliance (**D**) were measured by FlexiVent in response to increasing doses of MCh.

In order to confirm the results obtained by passive allergic sensitization and further investigate the role of IgE-mediated mechanisms in airway inflammation *in vivo* we used active (intraperitoneal) sensitization followed by intranasal allergen challenge with *Af*[38]. Wild-type and Lp-PLA_2_-/- mice received two injections of *Af/*alum (Vol: 1:1) one week apart, and were treated intranasally with *Af* 6 days after the second *Af/*alum injection. Negative control mice received *Af* challenge alone (Figure 5A). Sensitized and challenged mice had marked inflammatory cell influx into the airways, predominated by eosinophilic granulocytes, a hallmark characteristic of the allergic airway response. Although there was a trend towards an attenuated airway eosinophilia in Lp-PLA_2_-/- mice in comparison with WT mice, this difference did not attain statistical significance (Figure 5B).

Lung function was measured 24 (not shown) or 48 hours after *Af* challenge of sensitized mice (Figure 5C-D). The greatest changes in lung function in comparison with non-sensitized controls were measured 48 hours after *Af* challenge but there were no significant differences between the WT and Lp-PLA_2_-/- groups at either time points. The top lung resistance values (6.00±0.73 *v.s*. 6.72±1.51 cmH_2_O.s/ml) and dynamic compliance values (0.0097±0.002 *v.s.* 0.0118±0.002 ml/cmH_2_O) were obtained in response to 100 mg/ml MCh from WT and Lp-PLA_2_-/- mice, respectively.

**Figure 6:**
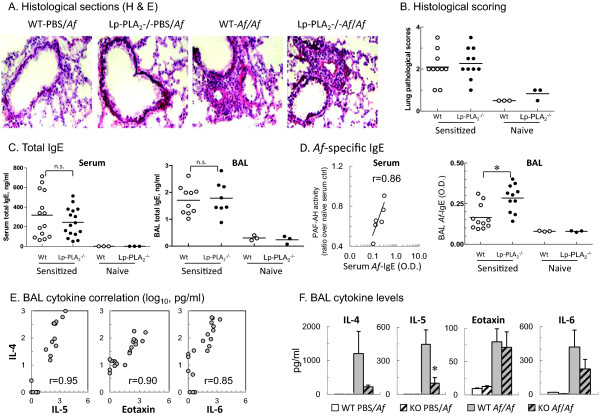
**Lp-PLA2-/- mice had similar degree of airway inflammation, circulating and local IgE but attenuated IL-4 and IL-5 release in the BAL fluid when compared with WT mice.** (**A**): Representative photomicrographs of H&E stained sections from formalin fixed and paraffin embedded lung tissue of each group. (**B**): Inflammation was semi-quantitatively assessed (median shown; n=3-11). The following inflammation score was used: (0-0.5): no infiltrates, intact lung structure. (1-1.5): <10 inflammatory cells/field. (2-2.5): Peri-bronchial/peri-vascular infiltrates. (3-3.5): peribronchial/vascular cuffing with parenchymal extensions. The entire tissue section was scored. (C-D): Total IgE and anti-Af specific IgE was measured by ELISA from serum and BAL samples of either non-sensitized (n=3) or Af-sensitized (n=11-16) mice. All animals received a single Af challenge. Maxisorp 96-well plates were used and IgE was detected by HRP-conjugated anti-mouse IgE. Data presented as ng/ml for total IgE with group median (**C**) or OD450 nm with group median for anti-Af specific IgE (**D**). The measurements were performed in duplicates. (**D** left panel): A strong positive correlation is shown between serum Lp-PLA2/PAF-AH levels (ratio over naïve serum ctr) and Af specific serum IgE levels in WT mice (but not Lp-PLA2-/- mice, not shown), sensitized and challenged with Af. (E-F): Cytokine and chemokine levels in cell-free BAL supernatant were measured by a Luminex assay. (**E**): Correlation of Th2-related mediators with IL-4 (on the y axis). The points represent log10 transformed individual values. (**F**): Cytokine profiles shown among 4 groups, n=3-7. Data are presented as mean ± SEM, pg/ml. Student’t test *p<0.05.

Lung H&E staining confirmed airway inflammation after *Af* challenge of sensitized mice with the inflammatory infiltrates localized in the peribronchial and perivascular area. Histological scoring revealed no significant differences between WT and Lp-PLA_2_-/- mice. Control unsensitized mice had only a few inflammatory cells (Figure 6A-B).

To determine if lack of Lp-PLA_2_ predisposes to heightened IgE production, ELISA assay was performed (Figure 6C-D). IgE in BAL and serum of mice sensitized and challenged with *Af* was significantly increased but there were no differences between WT and Lp-PLA_2_-/- mice. Anti-*Af* specific (but not total) immunoglobulin proportion (including IgG1, IgE) in the serum of sensitized/challenged WT mice showed a very strong positive correlation with the capacity of the serum to degranulate mast cells (r=0.702 and 0.841, respectively) as well as with the serum Lp-PLA2 levels (PAF-AH activity, r=0.920 and 0.860). None of these correlations were observed in Lp-PLA2-/- mice suggesting that presence of Lp-PLA2 may regulate proinflammatory events leading to increased immunoglobulin production. It was somewhat unexpected therefore that we found that *Af*-specific immunoglobulin levels (IgE, IgG1 and IgG2a) were generally greater in the allergen exposed Lp-PLA2-/- mice than in WT mice. The underlying mechanisms and the immune regulatory role of Lp-PLA2 in antigen presentation are currently investigated in our lab.

Luminex assay of BAL fluid showed that *Af* challenge of sensitized mice significantly increased IL-4, IL-5 IL-6 and eotaxin levels (Figure 6E-F). CCL17 (not shown) was also significantly induced while the Th1 cytokines IL-2, IFN-γ, TNF-α, and IL-17 (not shown) were produced at very low levels 48 hours after *Af* challenge of sensitized mice. IL-4 the central cytokine in eliciting the allergic airway changes correlated well with levels of IL-5, eotaxin and IL-6 (Figure 6E). Interestingly, lack of Lp-PLA_2_ did not increase expression of any of these proinflammatory mediators. Instead, the Lp-PLA_2_ deficient mice had attenuated release of IL-4 (81.5% decrease from WT levels), IL-5 (77.8% decrease from WT levels; p<0.05, n=7), eotaxin (10.6% decrease from WT levels) and IL-6 (46.5% decrease from WT levels) (Figure 6F). These results imply that allergen induced cell-mediated local responses were commensurate with the airway hyperresponsiveness measurements and that Lp-PLA_2_ deficiency did not enhance the allergic inflammatory changes. The significance and mechanisms behind the reduced BAL Th2-type cytokine production in the Lp-PLA_2_-/- mice will need further investigation. Systemic expression of the Th2 cytokines including IL-4 in the serum of mice was also studied using a bead based (CBA) assay. Cytokine levels in the serum however appeared to be very low indicating that levels of local cytokines may better reflect the extent of allergic immune response than those of in the systemic circulation. The finding of reduced IL-4 and IL-5 levels without reduction of IgE in the BAL fluid of the Lp-PLA2-/- mice is somewhat unexpected. However, such discrepancy may be explained by the complexity of the mechanisms leading to immunoglobulin production and involving antigen presentation, T cell-B cell interactions, B cell and plasma cell functions. Each of these mechanisms can have multiple regulatory pathways that are not necessarily dependent on Th2 cytokines. The role of Lp-PLA2 in these is virtually unknown and should be studied further.

PAF is a potent agonist that is highly active in the nM range. The fact that PAF-AH activity in serum was not completely eliminated could account for the relatively little differences seen between the inflammatory responses of Lp-PLA2-/- and WT mice. Lp-PLA2-/- mice show approximately 10 nM/min/ml residual PAF-AH activity in their serum, which may still be effective in models of inflammation. Indeed, this is an important finding because major criticism of clinical efforts to Lp-PLA2 antagonism was based on the fear from potential elimination of PAF metabolism resulting in increased PAF levels and susceptibility to and severity of allergic/anaphylactic events [19]. We believe that the cleavage of PAF is not the exclusive mechanism of action of this enzyme and our presented study supports this argument. Additional support for this is provided by a recent study in which treatment of mice with darapladib (an Lp-PLA2 inhibitor) inhibited serum IL-6 levels but did not alter serum PAF levels in a model of atherosclerosis [46]. The residual PAF-AH activity in the serum of LP-PLA2-/- mice indicates that PAF can be cleaved via additional pathways and therefore susceptibility to allergic inflammation may not be increased by the targeting of Lp-PLA2. This is demonstrated by our data showing that allergen induced inflammatory changes are not enhanced when Lp-PLA2 is eliminated in Lp-PLA2-/- mice.

There is limited knowledge on alternative PAF cleaving enzymes but it was shown for example that PAF can be degraded by an esterase secreted from group A streptococcus [55], an approach that could be used in future studies to verify the importance of PAF-related inflammatory pathways.

## Conclusions

This study investigated the effects of PAF-AH/Lp-PLA_2_ deficiency on IgE-mediated airway inflammation. We hypothesized that targeting this enzyme will enhance allergen-induced cellular inflammation involving PAF. Our results indicate that lack of PAF-AH/Lp-PLA_2_ in the serum of knock-out mice or inhibition of it with SB-435495 significantly reduced but did not completely abolish PAF hydrolyzing activity *in vitro*. PAF inhalation enhanced LPS-induced airway inflammation in WT and Lp-PLA_2_-/- mice to a similar extent. Sensitized WT and Lp-PLA_2_-/- bone-marrow derived mast cells matured similarly and released β-hexosaminidase upon stimulation with allergen or IgE crosslinking to a comparable degree. Wild type and Lp-PLA_2_-/- mice responded to passive or active allergic sensitization by significant and equal airway inflammation and hyperresponsiveness after *Af* challenge. There were no differences in the amount of total IgE levels in the *Af* sensitized WT and Lp-PLA_2_-/- mice.

Taken together, these results indicated that Lp-PLA_2_ deficiency does not increase local cell-mediated allergic immune responses or airway hyperresponsiveness in these models. Our study is the first in which absence of PAF-AH/Lp-PLA_2_ is investigated *in vivo* using gene deficient mice.

## Abbreviations

Af: Aspergillus fumigatus; BAL: Bronchoalveolar Lavage; CD: Cluster of Differentiation; DNGP: 1-decanoyl-2-(4-nitrophenylglutaryl) phosphate; DNP-IgE: Anti-Dinitrophenyl Immunoglobuline E; ELISA: Enzyme Linked Immunosorbent Assay; ES cell: Embryonic Stem Cell; HDL: High Density Lipoprotein; HEPES: 4-(2-hydroxyethyl)-1-piperazineethanesulfonic acid; i.n: Intranasal; i.p: Intraperitoneal; IACUC: Institutional Animal Care and Use Committee; LDL: Low Density Lipoprotein; Lp-PLA_2_: Lipoprotein-Associated Phospholipase A_2_; LPS: Lipopolysaccharide; MCh: Methacholine; OD: Optical Density; PAF: Platelet Activating Factor; PAF-AH: Platelet Activating Factor Acetylhydrolase; PBS: Phosphate Buffered Saline; RPMI: Roswell Park Memorial Institute medium; WT: Wild Type.

## Competing interests

Financial competing interests.

· In the past five years have you received reimbursements, fees, funding, or salary from an organization that may in any way gain or lose financially from the publication of this manuscript, either now or in the future?

Steven A. Sheardown is an employee of Takeda Cambridge Limited, 418 Cambridge Science Park, Cambridge, UK.

Stephen Wilson is an employee of GSK Laboratory Animal Sciences, GlaxoSmithKline, Stevenage, UK.

Colin Macphee is an employee of GlaxoSmithKline, Department of Vascular Biology and Thrombosis, King of Prussia, PA.

· is such an organization financing this manuscript (including the article-processing charge)? If so, please specify.

YES. GSK provided financial support in the form of a research grant.

· do you hold any stocks or shares in an organization that may in any way gain or lose financially from the publication of this manuscript, either now or in the future? If so, please specify.

Steven A. Sheardown is an employee of Takeda Cambridge Limited, 418 Cambridge Science Park, Cambridge, UK.

Stephen Wilson is an employee of GSK Laboratory Animal Sciences, GlaxoSmithKline, Stevenage, UK.

Colin Macphee is an employee of GlaxoSmithKline, Department of Vascular Biology and Thrombosis, King of Prussia, PA.

· do you hold or are you currently applying for any patents relating to the content of the manuscript? Have you received reimbursements, fees, funding, or salary from an organization that holds or has applied for patents relating to the content of the manuscript? If so, please specify.

NO.

· do you have any other financial competing interests? If so, please specify.

NO.

Non-financial competing interests.

· are there any non-financial competing interests (political, personal, religious, ideological, academic, intellectual, commercial or any other) to declare in relation to this manuscript? If so, please specify.

NO.

## Authors’ contributions

ZJ performed experiments on the animal models, participated in experimental design, lung function measurements, data assembly, analysis and manuscript writing. MF worked on the animal models, participated in experimental design, data collection and analysis. GR performed the in vitro mast cell culture. BK helped with the animal colony maintenance, animal models and tissue collections. IGR provided technical help with tissue collections, cell counts and data base setup. SAS participated in molecular biology and targeted ES cell generation during KO model development. SW did the blastocyst injection and management of mouse breeding & analysis during KO model development. CHM participated in generation of hypothesis, experimental design, data interpretation, manuscript writing. AH participated in generation of hypothesis, experimental design, data interpretation, manuscript writing and was responsible for the overall direction of work. All authors read and approved the final manuscript.
